# The New York Institute of Stomatology

**Published:** 1903-10

**Authors:** 

**Affiliations:** The New York Institute of Stomatology


### Reports of Society Meetings.

### THE NEW YORK INSTITUTE OF STOMATOLOGY.

A REGULAR meeting of The New York Institute of Stomatology was held on Tuesday evening, October 7, 1902, at the residence of Dr. J. B. Loucherty, 136 West Forty-eighth Street, New York City.

The President, Dr. J. Morgan Howe, occupied the chair, and called the meeting to order.

The minutes of the last regular meeting were read and approved.

*The President.*—Under "Communications on Theory and Practice," Dr. St. George Elliott has kindly agreed to present a report of some experiments with archite.

*Dr. Elliott.*—Before taking up this very interesting subject of archite, I would like to say that we have offered to us by the medical profession a new antiseptic, with which I have been experimenting to some extent. It has been largely recommended in the treatment of typhoid fever, but it appeals to us from its dental possibilities. It is thirty times as strong as bichloride of mercury, harmless, non-poisonous, and can be taken in five-grain doses internally. I have used it to a limited extent in the treatment of root-canals. It is one of the phenol products, and is called aceterone. It is made by Parke, Davis & Co. It is somewhat expensive, and is alloyed, so to speak, with an inert powder to reduce its strength fifty per cent. It is slightly soluble in water and decomposed by organic matter. I mention this because it seems to me a remarkable inconsistency in the literature of the subject. If it is rapidly decomposed by organic matter, how can it ever reach the intestinal canal intact?

In regard to archite, I commenced my experiments soon after the material was introduced, and was very much pleased with it. Its working characteristics were very pleasant. I noticed that it set very rapidly, soon became very hard, and was difficult to manipulate on account of this hardness. I have made a great many inquiries, and find there is a general dissatisfaction in regard to its use, as it does not stand well in the mouth. Its cohesion to the walls of a cavity is not as good as the ordinary phosphate, but with proper care, preparing the cavity as for amalgam, you get a fair or sufficient amount of cohesion. While it presents certain advantages in its translucency, taking color beautifully, and matching the color of the teeth very perfectly, it does not seem to have any particular value in regard to durability, and it was in this direction we had the greatest expectations. However, I believe certain qualities have been developed in the manufacture of this material which eventually may give us an ideal filling. Only two days ago it was necessary for me to take out an archite filling in a molar which I had put in about two months ago, and I had extreme difficulty in drilling it out. If it has that particular characteristic, and the manufacturers can overcome some of its defects, it will be very valuable. I tried a number of experiments as to how long it should be kept free from moisture. I found out, as one would naturally expect, that if it were mixed thin and placed at once in water, it never attained any great degree of hardness, and was very chalky in character; but if it were protected either by cellulose (which is celluloid in liquid form) or by paraffin, it did gain the maximum degree of hardness. Its penetration by coloring-matter—red ink particularly—was in accordance with this fact. If it were mixed thin and placed in ink, the ink penetrated rapidly—not as rapidly as some of the phosphates, but to an appreciable extent; but if it were allowed to harden for ten minutes, there was practically no penetration.

There is a feature in the mixing of archite which may be noticed also in phosphate: if you mix it slowly—it may be because there is a smaller amount of powder used—it will set slowly, sufficiently so for you to properly contour your filling; but if you mix it quickly, it is difficult to use, because of its hardness, setting so quickly.

There was so much dissatisfaction about it that I understood Smith & Son, of Pittsburg, who brought it out, had recalled it; but I have had circulars from them since saying that they had not recalled it, that some were satisfied with it, but it had not met with the approval they anticipated. They have since issued it in an improved form, and expect better results.

Subject passed.

The paper of the evening was then read by Dr. Edward H.

Angle, of St. Louis, entitled "Some Basic Principles in Orthodontia," the same being accompanied with illustrations on the screen.

(For Dr. Angle's paper, see page 729.)

HK

DISCUSSION.

Dr. S. H. Guilford, of Philadelphia, said that Dr. Angle had made such a fine exposition of the subject that it would be difficult to add anything to it. He wished to emphasize some of the points made by Dr. Angle and to refer to others upon which they differed somewhat. He expressed his pleasure at the growth of this branch of dentistry since he began practice, and his great interest in it. There are certain "basic principles" which should be emphasized,—that it is impossible for a practitioner to fill a tooth, make a plate, or correct a case of irregularity equally well, for the proper amount of skill necessary to accomplish this cannot be developed with a few cases.

Practitioners do not consider how much knowledge of dynamics and physiology and good reasoning power is required to work out the problems presented.

He believes that orthodontia should be made a specialty, and that those practising it should confine themselves to it as far as possible in order to develop the skill necessary.

A man should have a knowledge of the physiological characteristics of the teeth and the parts surrounding them in order to understand how force should be directed at different points to the best advantage. A man making this a specialty should also have an understanding of the law of harmony, and an artistic instinct to aid his judgment as to what is necessary in each case.

If a younger practitioner wishes advice of some one older, he should first work out the problem for himself and then submit the case and ask whether his plan can be improved and whether it is right or wrong.

Dr. Guilford said that he differed somewhat from Dr. Angle regarding extraction. He finds that in certain cases it is best to extract, and believes this is where individual judgment is of value. He does not advocate extraction in general, but cannot reconcile himself to the idea of never extracting. He mentioned two cases illustrating this idea: First, a boy of about fifteen with fairly good occlusion, with two exceptions, an upper cuspid standing out and no room for it in the arch. In the lower jaw, one of the second bicuspids was inlocked and more than half the space taken up by the crowding together of the other teeth. The boy's father was a large man and the boy favored him. Dr. Guilford stated to the patient's mother that it would be better to expand the arches so that there would be harmony when the face had attained its full growth; that extraction would have been positively harmful.

Second, a little girl apparently undersized; her mother and sister small; patient evidently to be like her mother. The occlusion was perfect, except that the lateral stood outside of the arch. After extracting the first bicuspid he moved the cuspid and lateral back, preserving the occlusion. The arches would have been too large for the face if they had been expanded. Dr. Guilford does not believe in hard and fast rules, and feels that we should be governed by circumstances, and where extraction would be right in one case it would be wrong in ten or twenty others.

Dr. C. F. Allan, of Newburgh, called attention to Dr. Angle's plea for good models, and exhibited some specimens of Dr. Angle's work in that direction.

Dr. G. S. Allan said that he had always looked askance at the idea of orthodontia being a specialty until he visited Dr. Angle's office, saw his apparatus, and watched the cases brought in to have their appliances attended to. He then became convinced that Dr. Angle was right that orthodontia should be practised as a specialty.

Dr. Allan thought that another point should be emphasized,—the fact that there is no appliance to be made; the apparatus is at hand for every case that presents, and has only to be adapted.

After thanking the essayist, Dr. Allan expressed pleasure that it had been possible for him to bring Dr. Angle before the Institute and give him the opportunity to present the subject in such a beautiful and masterful manner.

Dr. V. E. Barnes, of Cleveland, said that after constant use of the jack-screw and complicated fixtures the wonderful results attained with simple appliances was inspiring. Being dissatisfied with jack-screws, and feeling that there was a waste of time and energy, he had taken to the arch wire, and found his cases progressing two or three times as fast as before and with less annoyance to the patient. Dr. Barnes thinks that there is much promise in the Baker anchorage. He also stated that he hoped we had reached the dawn of a new era where extraction was to be the exception and not the rule. He believes that degeneracy and inheritance has been given too much credit as causes of irregularity.

Dr. Shaw, of Springfield, Mass., expressed his commendation of the views set forth by Dr. Angle.

Dr. J. Bond Littig said that the paper had been a revelation to him, especially regarding the facility with which the teeth were moved. He felt that the paper had been of vast benefit to those interested in the subject.

Dr. E. A. Bogue expressed his earnest thanks to Dr. Angle, and said that the importance of the occlusion of the first permanent molars had never been so clearly stated before in the Institute; indeed, he did not know where it had been brought out in print so clearly and concisely.

The first statement made of it that he knew of was in a paper of his own which he brought out in April before the American Medical Association.

The first permanent molar was erupted at the time when all the deciduous teeth were in the mouth. They were the posts on which the jaws were designed to rest during the shedding of the temporary and eruption of the rest of the permanent teeth.

He believes that the dentist's first duty is to see that these teeth occupy the proper relative positions towards each other. Then there would be very few cases that could not easily be regulated in all other particulars.

Dr. Leroy stated that although he had read much dental literature on the subject of orthodontia, Dr. Angle's remarks brought out the principles more clearly than anything he had seen on the subject. He thought that the cause of failure in much of the regulating was due to the fact that the men undertaking it did not understand the principles; that those undertaking regulating should bear this principle in mind; the two main points to be considered were the alignment of the first permanent molars and cuspid teeth.

Speaking of Dr. Angle's remarks about jumping the bite for young individuals under twelve years, Dr. Leroy mentioned two cases in which he had jumped the bite for patients of twenty.

Adjourned.

Fred. L. Bogue, M.D., D.D.S.,
*Editor The New York Institute of Stomatology.*

